# Multimethod study of a large-scale programme to improve patient safety using a harm-free care approach

**DOI:** 10.1136/bmjopen-2016-011886

**Published:** 2016-09-22

**Authors:** Maxine Power, Liz Brewster, Gareth Parry, Ailsa Brotherton, Joel Minion, Piotr Ozieranski, Sarah McNicol, Abigail Harrison, Mary Dixon-Woods

**Affiliations:** 1 HAELO, Salford Royal NHS Foundation Trust, Salford, UK; 2 Faculty of Health and Medicine, Lancaster Medical School, Lancaster University, Lancaster, UK; 3 Institute for Healthcare Improvement, Cambridge, Massachusetts, USA; 4 Data to Knowledge Group, School of Social and Community Medicine, University of Bristol, Bristol, UK; 5 Department of Social and Policy Sciences, University of Bath, Bath, UK; 6 Education and Social Research Institute, Manchester Metropolitan University, Crewe, UK; 7 Cambridge Centre for Health Services Research, University of Cambridge School of Clinical Medicine, Cambridge, UK

**Keywords:** patient safety, measurement, improvement programmes, quality improvement collaboratives, mixed-methods

## Abstract

**Objectives:**

We aimed to evaluate whether a large-scale two-phase quality improvement programme achieved its aims and to characterise the influences on achievement.

**Setting:**

National Health Service (NHS) in England.

**Participants:**

NHS staff.

**Interventions:**

The programme sought to (1) develop a shared national, regional and locally aligned safety focus for 4 high-cost, high volume harms; (2) establish a new measurement system based on a composite measure of ‘harm-free’ care and (3) deliver improved outcomes. Phase I involved a quality improvement collaborative intended to involve 100 organisations; phase II used financial incentives for data collection.

**Measures:**

Multimethod evaluation of the programme. In phase I, analysis of regional plans and of rates of data submission and clinical outcomes reported to the programme. A concurrent process evaluation was conducted of phase I, but only data on submission rates and clinical outcomes were available for phase II.

**Results:**

A context of extreme policy-related structural turbulence impacted strongly on phase I. Most regions' plans did not demonstrate full alignment with the national programme; most fell short of recruitment targets and attrition in attendance at the collaborative meetings occurred over time. Though collaborative participants saw the principles underlying the programme as attractive, useful and innovative, they often struggled to convert enthusiasm into change. Developing the measurement system was arduous, yet continued to be met by controversy. Data submission rates remained patchy throughout phase I but improved in reach and consistency in phase II in response to financial incentives. Some evidence of improvement in clinical outcomes over time could be detected but was hard to interpret owing to variability in the denominators.

**Conclusions:**

These findings offer important lessons for large-scale improvement programmes, particularly when they seek to develop novel concepts and measures. External contexts may exert far-reaching influence. The challenges of developing measurement systems should not be underestimated.

**Strengths and limitations of this study**

- The multimethod design enabled a holistic evaluation.
- The study reveals the impact of policy and structural turbulence on ability to achieve change in health systems.
- The importance of a rigorous development phase for improvement programmes, including significant investment upfront in measurement and data systems, was identified.
- The process evaluation of the first phase of the programme may have been biased towards those with more positive views.
- Independent data on clinical outcomes were not available, and the evaluation thus relied on data collected by the programme itself.

## Introduction

How best to ensure the safety of patients continues to challenge health systems worldwide.^1–3^ Recent years have seen multiple efforts to secure improvements. Some have multiple safety targets and seek generalised strengthening of organisational systems, processes and cultures,^4–6^ while others target specific areas of harm or practice.^7–9^ Whatever their form, improvement programmes typically measure outcomes one by one, with incidence for each—for example, central venous catheter bloodstream infections or unplanned readmissions to hospital—reported singly and separately, rather than in terms of how many harms each person suffered. Most also focus on specific, well-bounded healthcare settings and measure harms that are assumed to be attributable to the care provided in those environments.

Some (though not all) patient safety programmes have reported welcome successes in relation to specific harms. From the patient's perspective, however, a focus on single outcomes in well-bounded healthcare settings may be deficient, potentially obscuring individuals' experiences across pathways of care and their exposure to concatenations of multiple adverse events.^10^ Addressing harms singly also has other unintended consequences, including the reinforcement of disciplinary boundaries. Infection control nurses may, for example, work in isolation from tissue viability nurses with the same patients. Thus, a potentially more useful approach to safety might focus on the extent to which patients escape all possible harms and could thus be deemed to have experienced care that is ‘harm-free’. In this article, we report a study of a large-scale programme seeking to promote an innovative approach to harm-free care in England.

## The harm-free care programme

Run as part of the Department of Health's Quality, Innovation, Productivity and Prevention (QIPP) ‘Safe Care’ workstream,^11^ the programme was led by a dedicated national programme team and had three major goals:

- Develop a shared national, regional and locally aligned safety focus for four high-cost, high volume harms (venous thromboembolism (VTE), pressure ulcers, urinary tract infection in patients with urinary catheters and falls). These four harms were selected because they account for a large proportion of all avoidable injury to patients and share many underlying factors (eg, mobility, medication management, nutrition, hydration) relating to basic patient care, yet may involve trade-offs in managing risk.^1^ ^12^
- Establish a measurement system based on the principle that a new patient-centred measure that would ‘bundle’ harms into a single, composite score of harm-free care would bring new insights into harm rates, enable clinical teams to identify and recognise where problems lay and motivate local improvement.
- Deliver improved clinical outcomes, with a specific objective of ensuring that 95% of patients would be harm-free.

The programme did not seek to develop new technical interventions for managing the four harms nor to set targets, but instead sought to ensure that addressing the four harms together for each patient was identified as a priority for organisations, to support organisations and teams in implementing existing good practice in relation to the four harms, and to provide a well-founded means of surveillance, monitoring and feedback on harm-free care. It ran in two distinct phases. The first phase ran September 2010 to April 2012, including a 3-month preparatory period at the beginning (September 2010–December 2010) and a 6-month maintenance period at the end (October 2011–April 2012). This first phase sought to pilot an approach to measuring and improving patient safety, to support a cohort of organisations to implement and test it and ultimately to prepare the way for the subsequent use of the approach across all care settings in England. To achieve these aspirations, the national team undertook an intensive period of programme design, refinement of operational definitions, cycles of testing and learning and developing and modifying a data collection tool for harm-free care.

This tool, which came to be known as the National Health Service (NHS) Safety Thermometer, sought to enable collection of data that would be comparable at a national level and useful in local improvement work^13^ and that would balance accurate measurement and standardised definitions with straightforward data collection methods that did not burden staff. The design period was followed by work to implement the programme through regional and local partnerships (table 1), much of it organised through a voluntary quality improvement collaborative known as Safety Express.

**Table 1:** Safety Express key deliverables and review points for regions, determined in advance

Baseline assessment	Review 1	Review 2	Review 3	Final review
Safety Express phase reviews			Maintenance phase review	Incentivised phase review
Sept–Dec 2010	April 2011	Sept 2011	Sept 2012	March 2013
A named individual in each region to link into the national team, appoint a local team and link into the QIPP team. Identify areas of alignment and discourse between local, regional and national QIPP plans. Recruit 10 host organisations and ensure team composition included locality partners. Identify regional faculty for a Safety Express improvement collaborative. Field 100 people at learning session 1 of the collaborative.	Integration of the safe care plans into the regional QIPP plan. Ten teams of 10 participating in the collaborative. Participation in fortnightly WebEx meetings (regional leaders) Submission of monthly data using the NHS Safety Thermometer Faculty support (‘national’ and ‘regional’—national included subject matter experts, ie, in tissue viability/pressure ulcers and nutrition. Regional—leading clinicians and QI experts) to teams between learning sessions (WebEx/site visits/phone calls).	Submission of five case studies of ‘innovative practice’ to the national team. Submission of monthly data using the NHS Safety Thermometer from each organisation in the collaborative. Well-defined plans for scale up to the remaining organisations in the region, including plans to work collaboratively with commissioners. Identification of teams to put forward for national awards at a Summit event at the end of the pilot. Plans to publish the work.	All organisations in the region to have participated in the CQUIN for collecting NHS ST data monthly. Engagement with Clinical Commissioning Groups to raise awareness of ‘harm-free’ care programme and the NHS Safety Thermometer CQUIN (eg, attendance at the Safe Care work stream meeting for commissioners, attendance at CQUIN master classes in which the details of the CQUIN were explained to commissioners from each region). Review regional level data. Publication of the results of the QIPP Safe Care programme of work. Evidence of regional planning for delivery of improvement for the 2013–2014 CQUIN.	All organisations participating in the 2013–2014 CQUIN to aim to achieve 50% improvement in reduction of the four harms by March 2014. Evidence of the harm-free care programme in Trust's Quality Accounts and/or Trust Board reports. All CCGs commissioning harm-free care locally. All CCGs and organisations to have systems in place to embed ‘harm-free’ care into contracts and to embed into the new NHS and social care structures. Evidence of support to assist organisations who have not achieved 50% improvement.

CCGs, Clinical Commissioning Groups; CQUIN: Commissioning for Quality and Innovation; NHS, National Health Service; NHS ST, NHS Safety Thermometer; QI, quality improvement; QIPP, Quality, Innovation, Productivity and Prevention.

Use of the collaborative model^14^ was based on the theory that it would facilitate rapid shared learning and the mobilisation of collective cross-multidisciplinary action.^15^ Consistent with the BreakThrough Series collaborative approach,^16^ Safety Express involved three learning events where participants across the regions came together, and action periods during which participants were asked to implement improvement activities (eg, setting up data collection systems and implementing Plan-Do-Study-Act cycles). Participants were recruited through the 10 strategic health authority (SHA) regions then extant in the English NHS. Each region was asked to engage 10 participating organisations serving a local population, and each of these organisations was asked to ensure that 10 staff members (primarily front-line clinicians) attended the learning events and that they tested the NHS Safety Thermometer (NHS ST) in a relevant caseload. The work of clinical teams in undertaking these activities was supported by the regional and national teams, as well as by online resources and detailed guidance on good practice interventions and on how to submit and interpret local data. Participating organisations were asked to collect data on four wards (acute) or on their caseloads (non-acute) on 1 day per month using the NHS Safety Thermometer and to submit it to a central data collection facility. A 6-month maintenance period during which organisations were asked to continue submitting data followed the completion of Safety Express in September 2011. Some support, albeit limited, was available to organisations on request during the maintenance period.

The second phase of the programme ran April 2012 to March 2013, when it expanded beyond the original participants to include all settings providing care for NHS patients in England. An important characteristic of this phase is that financial incentives through the Commissioning for Quality and Innovation (CQUIN) mechanism were offered to all NHS organisations in England to submit data on 100% of patients on 1 day per month using the NHS Safety Thermometer. Only limited improvement support was available: the collaborative did not continue, though access to online resources remained, and some limited support was available from the NHS Institute for Improvement and Innovation up to March 2013. Some locally organised (not nationally coordinated) support activity also took place.

## Study aim

The available evidence suggests that large-scale programmes may offer some important advantages over single-organisation efforts^17^ by supporting the infrastructure for improvement, including the development of well-founded interventions and data systems^18^ as well as activating the social conditions, peer-norming effects and shared learning most likely to foster change^15^ ^19^ ^20^ and enabling change at scale. A growing body of evidence now points to the features essential to the success of such programmes, including shared goals among participants, clinician engagement, clinical champions and the importance of well-designed, theoretically sound interventions.^5^ ^19^ ^21^ ^22^ However, large-scale improvement programmes continue to show a mixed picture of success, with many reporting disappointingly modest (or no) improvements in implementation of evidence-based interventions in practice.^23^ ^24^ These findings suggest that much remains to be learnt about these complex interventions,^25^ for example regarding contextual influences,^26^ programme design and implementation,^27^ measurement^28^ ^29^ and sustainability beyond project timelines.^30^

With the aim of addressing these gaps in knowledge and improving the evidence-base for future large-scale improvement programmes, particularly when they involve novel approaches and measures, our study sought to assess how far the harm-free programme met its three aims and to identify and characterise the influences on the achievement of these aims.

## Methods

We conducted a multimethod evaluation ‘wrapped around’ the programme,^31^ rather than a research study that set out to test specific hypotheses. During phase I of the programme, we used a combination of data collected by the programme itself and an independent process evaluation. The programme data included an analysis conducted by the national team of the extent to which regional strategy was aligned to national goals, information on the number of organisations that were submitting data on the four harms and the data on clinical outcomes (the four harms) submitted by participating organisations. As part of a wider study of quality and safety in the NHS,^32^ we also conducted a concurrent process evaluation of Safety Express (the quality improvement collaborative that ran during phase I), using interview, observational, questionnaire and documentary data. The process evaluation was used as part of a convergent design directed towards obtaining different but complementary data and thus developing a more complete understanding of the programme at multiple levels.^33^ Approval for the process evaluation was obtained from an NHS Research Ethics Committee (REC). Signed consent was obtained for interviews.

For reasons of resource, only the programme data on submission rates and clinical outcomes submitted by the participating organisations were available for phase II. The organisations submitting data changed over time (especially between phases 1 and 2), leading to denominators that increased in size and diversity over time. Twelve organisations submitted data consistently from January 2011 to March 2013, and these were subject to subgroup analysis.

All quantitative data were collected and analysed by the programme team; all qualitative data were collected and analysed by an evaluation team independent of programme team (tables 2 and 3). The data were analysed separately and then synthesised thematically.^34^

**Table 2:** Quantitative data

Programme goal	Data	Analysis
*Develop a shared national, regional and locally aligned safety focus*, assessed through inclusion of the four harms in each region's system-level strategy plans and QIPP improvement programmes.	Each region's progress extracted from plans and mapped on four occasions using a categorical rating scale, used to assess achievement of programme goal of a shared national, regional and locally aligned safety focus for the four harms.	A judgement was made by the programme team to determine whether the region was achieving four or more of the milestones (green), two to three (amber) or one or less (red).
*Develop a shared, national, regional and locally aligned safety focus*: participation in the collaborative, delivery of the required programme outputs and NHS Safety Thermometer data collection.	The number of participating organisations and the number of attendees each region sent to each learning session in Safety Express was recorded, used to assess achievement of goal of a shared national, regional and locally aligned safety focus for the four harms.	Count data displayed as descriptive statistics and percentages.
*Establish a measurement system*, assessed by tracking number of sites submitting data over time.	Number of organisations submitting data on the four harms.	Description.
*Deliver improved clinical outcomes*, assessed by determining absence of all four harms at the individual patient level	For each patient, data were collected by local clinicians on four outcomes (pressure ulcers, falls, urinary tract infection in patients with urinary catheters, and VTE) and submitted using the NHS Safety Thermometer, used to monitor progress towards the programme of improved clinical outcomes. To allow for variation in organisations submitting data over time, two cohorts were formed: Data from acute patients from the initial, phase I Safety Express organisations consistently submitting between January 2011 and March 2013 Data from acute patients from all organisations submitting at any time between January 2011 and March 2013.	The composite measure of harm-free care was plotted over time using a control chart. To take account of overdispersion, due to the large sample size, a P′ control chart was used. Standard control chart rules were applied to indicate special and common cause variation and when a shift in the average occurred. Statistical analysis was performed using R-2.15.1 for Windows (http://cran.r-project.org/bin/windows/base/old/2.15.1/).

NHS, National Health Service; QIPP, Quality, Innovation, Productivity and Prevention; VTE, venous thromboembolism.

**Table 3:** Process evaluation (phase I only)

Method	Data	Analysis
*Semistructured interviews*. A prompt guide was used to elicit experiences and views of the programme from stakeholders who were purposively sampled to represent different constituencies (eg, national, regional and local). Participants were recruited through email coordinated by the national team. Theoretical sampling was not possible due to the nature of recruitment; instead all those who agreed to be interviewed over a defined time-frame were interviewed. It was not possible to assess theoretical saturation formally. Interviews were audio-recorded and transcribed.	Interviews with 7 QIPP national team members, 6 local coordination leads and 11 programme participants. The local programme coordination leads were more senior nursing staff, located in 4 of the 10 Strategic Health Authorities. The programme participants interviewed were mainly nursing staff with responsibility for clinical governance, tissue viability or patient safety and were based in 8 of the 10 regions. These data were used to assess influences on the programme's achievement of its goals of shared goals and establishment of a measurement system.	Analysis was based on the constant comparative method, facilitated by NVivo software.^35^ ^36^ Open codes were generated through close reading of transcriptions. Reflection and interpretation were used to produce a higher level of abstraction and thematic categories. Coding of transcripts was supported by NVIVO V.8 software.
*Observations*. Observers took detailed field notes and held de-brief sessions, which were audio-recorded and transcribed.	Ethnographic observations to assess the experience of participating in the programme were conducted at Six Safety Express learning events.	As above
*Survey*. On the basis of the observational and interview data, an online survey was developed and circulated to all learning event participants and through email contact channels. Covering implementation of the programme, data measurement and organisational involvement, the majority of the 40 survey questions were multiple-choice or Likert scale, with four free-text questions used to elicit more in-depth responses.	The survey received 157 anonymised responses; because of the method of email distribution, it was not possible to calculate a response rate. A diverse selection of respondents completed the survey (table 4), reflecting those participating in the project. These data were used to assess influences on the programme's achievement of its goals.	Descriptive analyses of the survey data, with free-text responses coded using content analysis.^37^
*Documents*. Project documentation and key policy documents were purposively sampled.	∼20 relevant documents, including policy materials, were collected from the programme team and from QIPP and other websites. These were used to gather information about the programme and possible contextual influences.	Review and summary.

QIPP, Quality, Innovation, Productivity and Prevention.

**Table 4:** Survey respondent characteristics

Site characteristic	Descriptor	Survey respondents* (%)
Organisation (n=133)	Acute trust	63.9
	Community trust	24.1
	Mental health trust	2.3
	Primary care trust	7.5
	Strategic Health Authority	6.0
	Other	4.6
Staff level banding† (n=134)	Bands 1–4	0.7
	Bands 5–6	10.4
	Bands 7–8	61.9
	Above Band 8	28.4
	Other	2.1
Regional cluster (n=134)	North	26.9
	Midlands/East of England	26.9
	London	6.7
	South East Coast	9.7
	South Central	21.6
	South West	6.0
	Prefer not to say	6.0

*Some respondents chose more than one option to describe their organisation, banding, and region.

†Most jobs in the NHS are covered by the AfC pay scales. This covers all staff except doctors, dentists and the most senior managers. The AfC job evaluation system determines a point score, which is used to match jobs to one of the nine pay bands and determine levels of basic salary (ref: http://www.nhscareers.nhs.uk/explore-by-career/nursing/pay-for-nurses/).

AfC, Agenda for Change; NHS, National Health Service.

## Results

**Figure 1:**
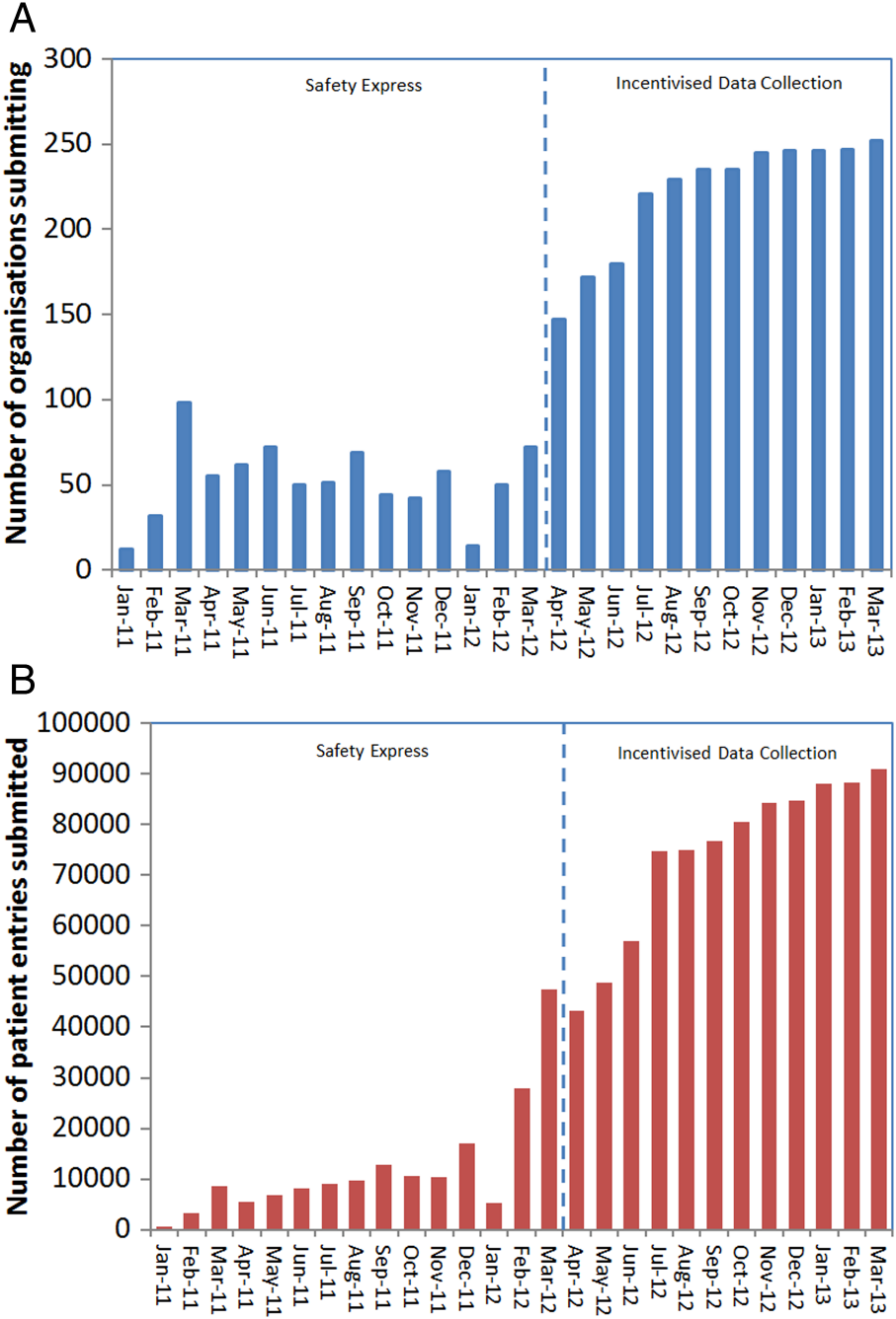
(A) Number of organisations submitting data over time (NHS Trusts submitting NHS Safety Thermometer data over time, from the start of the Safety Express programme (‘phase I’) through to end of the first period of incentivised data collection (‘phase II’). Bar height represents the total unique NHS Trusts submitting within the month. In January 2011, 12 organisations submitted. In March 2013, this had risen to 252 organisations). (B) Number of patient entries submitted over time (Number of individual patient-level entries submitted to the NHS Safety Thermometer over time from the start of the Safety Express programme (‘phase I’) through to end of the first period of incentivised data collection (‘phase II’). Bar height represents the total patients submitted within the month. In January 2011, 712 patients were surveyed and their data were submitted against the ‘harm-free’ Care measure. In March 2013, this was 98 372 patients).

The process evaluation, which focused on phase I only, involved 24 interviews, 157 survey responses (table 4), 48 hours of observation and around 20 documents. Data on clinical outcomes were submitted by participating organisations over both phases on the programme, though the composition of the contributing organisations and the consistency with which individual organisations submitted data varied over time (Figure 1A, B). Quotations are numbered to indicate different participants and preserve anonymity.

### Achievement of programme aim 1: develop a shared national, regional and locally aligned safety focus for the four harms

Evidence on the development of a shared national, regional and locally aligned safety focus for the four harms during phase I was assessed through an analysis of the plans that regions submitted to the national programme team. A mixed picture emerged. Substantial variability (table 1) was evident in how well the regions' plans were aligned with those of the national programme. Only 2 of the 10 regions' plans were rated as ‘green’ on the rating scale by September 2011 (almost 9 months after the start of the programme) and only one organisation maintained this for over a year (table 5).

**Table 5:** Regions’ participation in the collaborative and alignment with programme goals

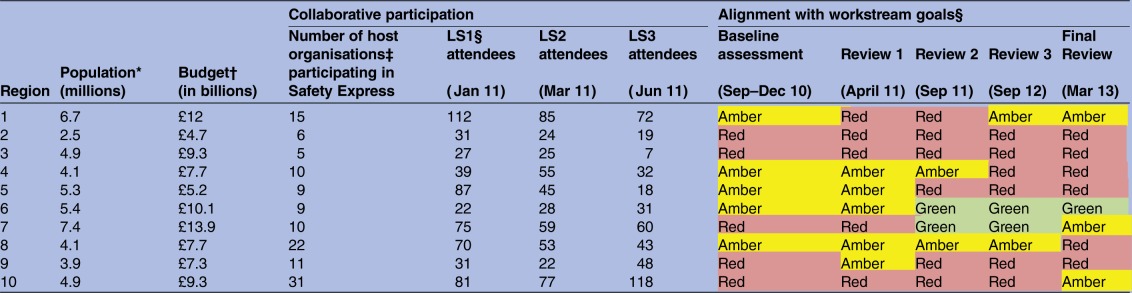

All 10 regions signed up to participate in the Safety Express collaborative, but only 2 were able to reach the goal they were set of recruiting 10 participating organisations. Instead, regions recruited between 5 and 31 organisations. No site was able to consistently provide 100 participants at each learning event (the numbers attending ranged from 22 to 118), meaning that the goal of enrolling 1000 front-line clinicians was not reached. In seven regions, attrition occurred in the number of delegates attending the learning events as the programme progressed.

Interviews showed that many (though not all) participants saw the principles underlying the programme as attractive, useful and innovative. Much support was expressed for the programme principle of taking a holistic approach to harm: almost two-thirds (64%) of survey participants strongly agreed or agreed that the four harms chosen were the most important for their organisation to address, and interview participants were also generally positive about the approach to harm-free care. Survey and interview data suggested that participants generally valued the collaborative features of the programme, with the learning sessions and encouragement from the national team seen as particularly useful. Observations at the Safety Express learning sessions found that participants demonstrated considerable enthusiasm and that the sessions helped to build relationships and share learning, ideas and practical tools.

I mean we could bounce ideas off them, say we have thought about this, is anybody else doing something similar who we can talk to? So they have got that information to signpost us. (Learning session participant I-05)

However, the ambition of the programme daunted some participants. Just under half (44.6%) of survey respondents reported that the programme was greeted with ‘initiative fatigue’ in their organisation. Though nearly three-quarters (73.2%) reported that achieving ‘harm-free’ care was a realistic goal for the NHS, just over a third (34.8%) thought their organisation was close to attaining it. Translating the enthusiasm generated by collaborative activities into local action remained a challenge for many.

The ethos of it is obviously just what it should be, but how achievable it is I am not sure. (Learning session participant I-06)

Brilliant for networking and we all left feeling positive […] it was the sustainability following the events [that was] difficult. Because obviously you leave the room full of ideas and you go back to your everyday work and… it's very difficult to keep it going, I have to say. (Learning session participant)

**Figure 2:**
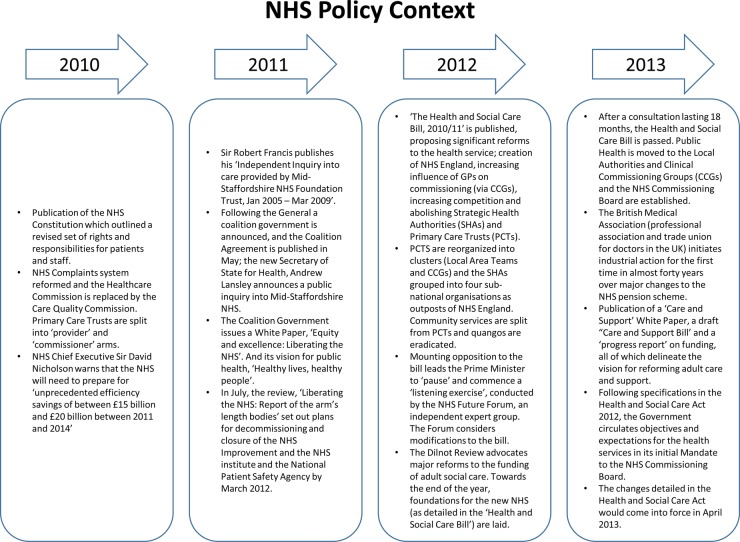
Timeline of key political and policy events 2009–2012. GPs, general practitioners; NHS, National Health Service.

The most profound influence on the ability of regions and organisations to engage with the programme appeared to be the context of extreme policy turbulence and structural change. Documentary analysis of the 2009–2012 policy context (figure 2) identified the transformations of the NHS architecture associated with the Health and Social Care Act (2012), with the effects evident before (in anticipation of) and after the passing of the legislation. Alongside many changes, a new national commissioning board was created (NHS England) and the 10 Strategic Health Authorities were replaced by 4 regional offices of NHS England. The national bodies that had supported system change were decommissioned (the NHS Institute in March 2013 and the National Patient Safety Agency in June 2012). Loss of senior leadership at the national and regional level contributed to voids of coordination and communication during the programme. Interviews with the programme showed that in all but one region, the problems faced in delivering the programme caused by external and internal turbulence necessitated implementation of a recovery plan and the establishment of direct communication between the national team and the participating teams rather than through the regions.

### Achievement of programme aim 2: establish a measurement system to understand the burden of the four harms

The programme largely succeeded in its aim of establishing a measurement system, but interviews and observations showed that the process of its development was effortful and it continued to generate considerable controversy throughout the programme.

Interviews, documents and observations found that a prototype of the NHS Safety Thermometer data collection tool was developed by the national programme team during the design period of phase I and refined iteratively thereafter. Intended to be used by front-line staff, who were asked to collect data on the four harms by reviewing patients' records and examining and speaking to the patient harms,^38^ the tool enabled entry of data through an online spreadsheet. It provided instant data display for the participating clinical teams and, through a merge function, supported aggregation to give whole organisational, regional and national data sets. Though rates of each of the four harms could be viewed separately, a novel feature of the NHS Safety Thermometer was its ability to generate a composite measure of ‘harm-free’ care to indicate the proportion of patients who had not experienced any of the four harms.

During Safety Express, the 10 regions were asked to coordinate collection of data using the NHS Safety Thermometer from 10 organisations from their region. Each of these 10 organisations was asked to collect data and submit on four wards (acute) or caseloads (non-acute) on 1 day per month. In interviews, many Safety Express participants saw the NHS Safety Thermometer as innovative, providing a useful and valuable data set that could be used to drive improvements and provide evidence of progress. They reported that the tool had several advantages in comparison with some available methods of measurement, including the potential that the tool provided for intervening and improving care on the spot. Some participants reported that working across all four harms helped to avoid duplication, both of data collection and effort.

The sheer number of nurses that have said: what is fabulous about it is that it means that I can improve patient care while the patient is right here, still in the bed and still when I can do something about it […] When I did the Safety Thermometer it was clear the [patient] had not had a VTE assessment done so I got the junior doctor to do it for them. (National team member I-22)

My understanding was that [the harms] selected themselves really because they were the biggest category of avoidable harms in healthcare and from that point of view I think they were the right ones to focus on. (Local organiser I-05)

Some participants (including around 20% of survey respondents) commented on the value of having shared definitions and being able to collect comparable data on harms across organisations.

The consistent approach across the country so we measure apples and apples. (Survey)

[NHS Safety Thermometer] is the first time that we have actually been nationally able to measure something in the same way to the same definition… I don't think that has happened in anywhere in Europe. (Local organiser I-15)

However, developing and deploying the NHS Safety Thermometer was not without substantial challenges. The time required to develop the tool was lengthy, much greater than the programme team had initially anticipated; modifications were still being made to the data collection instrument late in 2011, almost 11 months after the start of the collaborative. Questions and disagreement about the inclusion of the harms and their exact definition dogged the development of the programme, in particular, as shown by observations and interviews, by introducing delays while consensus was sought. This was particularly true of the inclusion of the measure relating to urinary tract infection in patients with urinary catheters, which some participants disputed or reported was unclear. Over 1 in 10 (11.8%) of survey respondents felt that the inclusion of this measure in the programme was not soundly based in scientific evidence, compared with just 2% feeling that there was no scientific basis to include VTE.

I think the other thing with falls and pressure ulcers is that there are quite clear definitions that everyone agrees on. For catheter associated UTIs it has not been the same… That has created quite a lot of confusion. (Local organiser I-15)

Because of some of the questions around the measurement piece, because of the questions around—well what does the definition for UTI look like in my organisation compared to yours? Very valid conversations but nonetheless quite stalling. (National team member I-18)

Some participants, for example in the sessions we observed as well as in interviews, expressed a very strong view that a national measurement strategy was neither useful nor appropriate. Organisations and individuals were often already using their own local definitions of some or all of the four harms and had established methods of data collection and data display (though these were largely from incident reporting systems). Participants did not always demonstrate consensus that the four harms chosen by the programme were the most important focus for improvement efforts in their own organisations.

The difficulty with it is that if we've already got a system and a process in place in some organisations to measure what they're doing against falls, pressure ulcers, whatever, individually, the link hasn't been there. (Local organiser I-24)

The number of updates to the tool over the course of Safety Express in response to feedback caused some frustration among participants, who did not always appreciate the developmental nature of the first phase of the programme. Participants also reported, in interviews and in the survey, that the tool was not as easy to use as intended. The extent to which data collection would need to be supported was initially underestimated by the national team; some months in, they reported that it became clear that there was a skills gap in relation to measurement in many participants, who were often inexperienced in collecting or using data for improvement. Documents and interviews showed that they produced a suite of materials to support learning and implementation and delivered a series of learning workshops across the country on measuring improvement, including technical capability (actual use of the data tool). In the survey, half (50.0%) of respondents described the tool as ‘straightforward’, but nearly half (44.3%) felt that data collection was a major burden. Some teams struggled to integrate the new data collection into their existing practice.

It's time-consuming. It's another thing that a clinician has to do. (Learning session participant I-01)

Around a third (32.6%) of survey respondents questioned the reliability of the data collected, indicating that they believed that it was ‘vulnerable to “gaming” by organisations trying to look good’ and that the data were not comparable across organisations. One problem was that the NHS Safety Thermometer asked data collectors to record whether the harm was ‘old’ or ‘new’ depending on when it occurred. Staff reported that this was a problem because of the way the tool seemed to obscure where and how the harm had occurred and opened up the possibility of blame.

But because it went down on our record, it looked as though it was ours even though it goes down as an old or a new, when you put those together it looks as though—oh look, they have got pressure ulcers. (Learning session participant I-06)

Substantial variability was evident in the extent to which organisations used the NHS Safety Thermometer during Safety Express (figure 1A, B). One region did not submit any data. In the first month, only 12 acute organisations submitted data, making 712 patient-level entries. Rates of organisational participation and data submission increased thereafter, with 140 organisations submitting data at least once and an average of 60 organisations contributing data every month throughout the collaborative. A total of 52 309 patient-level line entries were made during Safety Express. A majority (71%) of monthly submissions contained at least 30 patients and 84% achieved at least 20 patients. Data from hospital settings accounted for 90% of all data submitted, with the remainder from non-acute settings including 3% from the patients' own home, 2% from nursing homes and 5% from other settings. Within hospitals, 50% of the settings chosen by participants for testing in hospitals were medical wards.

During the second, incentivised data collection phase of the programme, the number of organisations contributing data increased dramatically: 719 organisations used the NHS Safety Thermometer during 2012–2013 (146 acute, 573 non-acute). This resulted in a large increase in patient entries into the data set: 1 882 558 patient entries (figure 1B). Diversity in the kinds of organisations contributing data also increased during 2012–2013, with particular growth in the proportion of patients from non-acute settings. Of the non-acute, 136 were independent provider sites and 217 were nursing homes. During this period, 58.3% of data were submitted from hospital settings, 7.8% from the patients' own home, 2.3% from nursing homes and 31.6% from other settings.

### Programme aim 3: deliver improved outcomes

**Figure 3:**
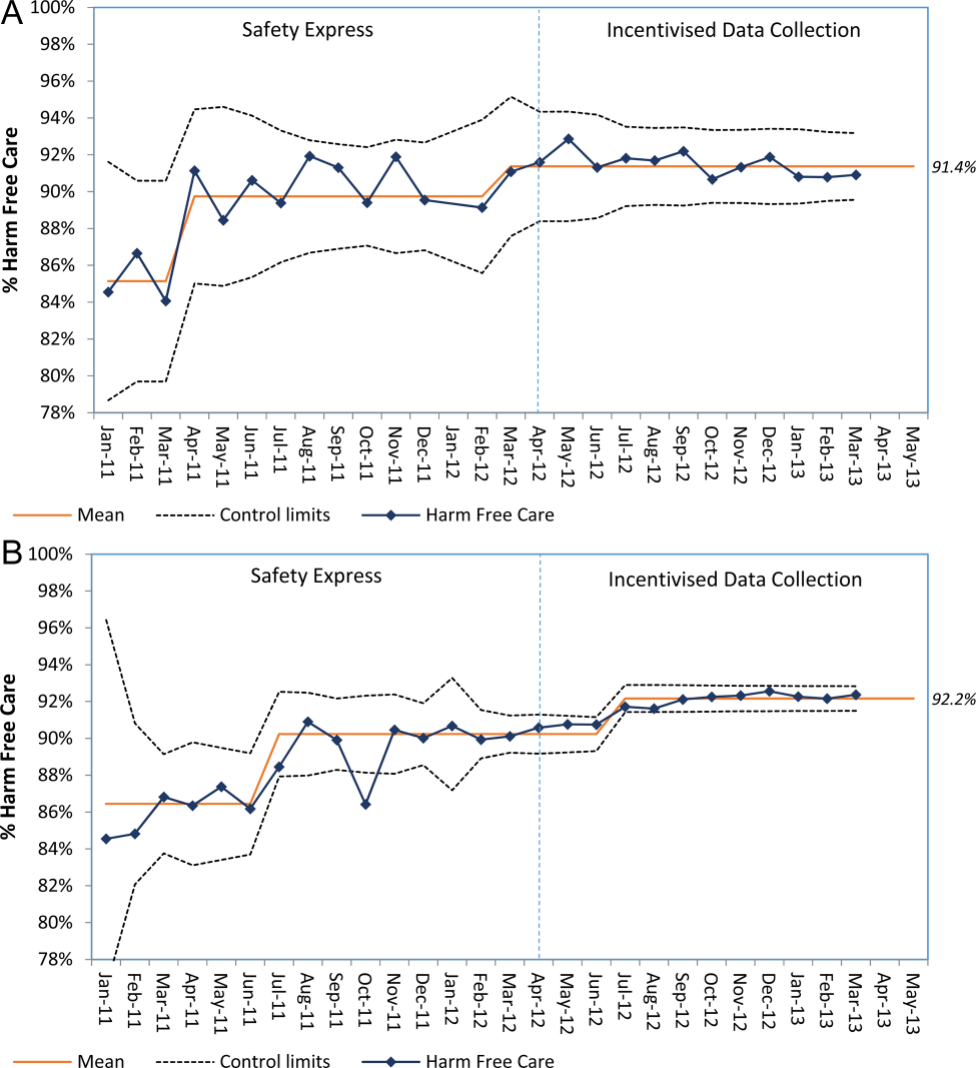
(A) Per cent harm-free care over time for patient entries submitted from the initial Safety Express (‘phase I’) cohort in January 2011 over time, until the end of the incentivised data collection period (‘phase II’) plotted as a P prime (P′) chart. (P′ chart showing per cent of patients from the initial cohort of Safety Express (‘phase I’) organisations experiencing harm-free care as defined by the NHS Safety Thermometer, presented over time. These data are plotted as a P′ chart; a type of control chart used for time-series data with a large denominator. Individual data points represent the % of patients in the cohort who received harm-free care each month; in January 2011, this was 85.1%. In March 2013, this was 91.4%. Control limits are used to apply control chart rules to detect special cause. The original plot of these data highlighted three distinct phases, indicated by the readjusted mean line). (B) Per cent harm-free care over time for patient entries from all submitting acute care trusts over time, from the beginning of the ‘Safety Express’ period (‘phase I’) to the end of the incentivised data collection period (‘phase II’) plotted as a P′ chart (P′ chart showing per cent of patients experiencing harm-free care (as defined by the NHS Safety Thermometer) while an inpatient in an acute bed, at any submitting NHS Trust, presented over time. Similar to (A), these data are plotted as a P′ chart. Individual data points represent the % of patients who received harm-free care each month; in January 2011, this was 86.5%. In March 2013, this was 92.2%. Control limits are used to apply control chart rules to detect special cause. The original plot of these data highlighted three distinct phases, indicated by the readjusted mean line).

The extent to which the programme met its aim of delivering improved outcomes was difficult to assess, given variability in the number and consistency of organisations submitting data over time. Control chart rules were used to interpret data on harm-free care over the two phases of the programme in the specific initial Safety Express subgroup of 12 organisations who were the first to join (figure 3A) and, separately, all organisations (including the initial Safety Express subgroup) (figure 3B).

The initial Safety Express subgroup organisations all reported data consistently over time. The proportion of harm-free care reported by these organisations rose from 85.1% in January 2011 to 89.7% in April 2011 during Safety Express. This increased further to 91.4% by March 2012 and remained stable up to March 2013 (throughout the incentivised data collection phase) (figure 3A). The proportion of patients who were deemed ‘harm-free’ in this subgroup did not reach the goal of 95%.

In all submitting trusts (including the initial Safety Express subgroup), the proportion of acute patients reported as receiving harm-free care rose from 86.5% January 2011 to 90.2% by July 2011 during Safety Express. This increased further to 92.2% in July 2012, and stabilised thereafter, during the incentivised data collection phase (end of March 2013) (figure 3B). Again, the 95% aspirational goal was not achieved.

## Discussion

This multimethod study of a large-scale, two-phase improvement programme using an innovative approach to harm-free care adds to the growing body of evidence on large-scale programmes as a means of securing change in healthcare. We set out to assess the extent to which the harm-free care programme met its aims and the influences on the achievement of those aims. We found that the programme struggled in developing a shared national, regional and locally aligned focus for the harm-free care concept during phase I, with policy turbulence a major influence in frustrating goal achievement. The goal of establishing a measurement system for harm-free care was achieved, but in the face of considerable challenge. Whether the third and final goal of improved clinical outcomes was achieved proved difficult to determine. These findings offer valuable learning about the design and conduct of large-scale quality improvement programmes in healthcare. First, this study illustrates the importance of significant upfront investment when launching new data collection tools based on novel concepts, especially when such tools seek to standardise the measures used across diverse settings. Second, it suggests that engagement in voluntary efforts such as quality improvement collaboratives may be contingent on relatively stable organisational and broader institutional contexts: participation and engagement in Safety Express remained patchy throughout its history. It was not until broader structures had settled, and a financial incentive for data collection was introduced in the second phase of the programme, that the reach and consistency of data submission improved. Third, this study illustrates the challenges in interpreting evidence relating to large-scale improvement. There is some indication that the proportion of patients experiencing harm-free care increased over the both phases of the programme, but trends over time in the aggregate submissions must be interpreted cautiously since the same organisations did not submit consistently over time nor did those who were submitting do so consistently, and case-mix varied over time.

One potentially tempting conclusion from this study is that the first phase of the programme was unnecessary since improved consistency of data submission did not occur until the second phase, which financially incentivised data collection. This second phase also saw possible improvements in clinical outcomes, even though little improvement support was available. Such a conclusion might suggest that future efforts to secure improvement should focus primarily on financial incentives, bypassing the messier and more uncertain path of voluntary, collaborative cooperation. But such an argument neglects the important developmental role played by the first phase. Without this, the second phase might have been foundered.

The developmental role of the first phase was especially critical in developing the NHS Safety Thermometer. Though quality improvement projects are known to be prone to measurement and data collection problems of various kinds,^30^ ^39^ ^40^ the challenges in developing measures and securing legitimacy are seldom reported. The concepts behind the NHS Safety Thermometer were novel, emphasising a patient-centred approach that required rethinking of traditional metrics and methods of data collection and display. Significant technical and social innovation was required to maximise the chance that the data would be regarded as credible while minimising the risk that data would be too irksome or burdensome to collect.^41^ Despite the level of investment and testing, some concerns about consistency, relevance and fairness endured among those submitting data, as has been found elsewhere.^42^

The first phase of the programme may have been important in developing approaches, definitions and tools, but less clear was the success of the collaborative model in securing change. Though the harm-free care concept was broadly recognised by Safety Express participants as an original and ingenious way to think about patient safety, none of the regions met the engagement metrics; ability to engage was adversely affected by contextual influences, including massive system instability that contributed to distraction, diminished energy and voids of leadership.^43^ It is also likely that the number of participants was too low to achieve the necessary momentum in an area the size of England. Further complicating engagement was the variation that existed between regions and between organisations in their approach to implementation. Better understanding of such variation might have enabled the national programme team to undertake a baseline assessment and codesign a bespoke programme with each locality. These findings affirm earlier evidence,^14^ ^39^ ^44^ indicating that quality improvement collaboratives may have some distinctive strengths but are far from a straightforward solution. It adds to this evidence in demonstrating that the potential of collaboratives may be heavily contingent on their political, economic and social contexts. Simply put, though they may have advantages over more coercive methods for making change,^45^ their success is likely to depend on a supportive outer context. Better understanding of how and when collaboratives are the right approach is an especially important goal given the known risks and limitations associated other means of achieving change, including those associated with use of financial incentives.^46^

A limitation of our study is that it was not possible to conduct a process evaluation of the incentivised data collection phase. This means that it is not easy to identify the mechanisms that might have contributed to the possible improvements in proportion of harm-free care that appear to have coincided with the introduction of the data collection requirement. One possibility is that the improvement observed was part of secular trend that was occurring anyway.^47^ Another is that the observed improvement is simply an artefact of the data collection process; as data collection expanded, the case-mix became more diverse and included a higher proportion of patients at lower risk of the four harms. A further possibility the introduction of financial incentives encouraged some form of gaming,^48^ though there is no direct evidence of this. Finally, it is possible that the observed change was real: that clinical teams did use the NHS Safety Thermometer as intended, recognising the value of a harm-free approach and using the data displays to identify where practice was falling short and making changes. Such an interpretation is consistent with the general observation that data plus feedback can act as an intervention, revealing unwarranted variations in practices, processes and outcomes and helping to inform targets for improvement.^49^ ^50^

Limitations of this study include its reliance on clinical outcome data reported to the programme by the participating sites: the data were not independently collected, nor was it possible to engage in verification or validation exercises. We interviewed all those who volunteered and sought disconfirming evidence where possible, but it is possible that those interviewed were primarily those with more positive views, since those participating in the collaborative were, almost by definition, more engaged. The online survey did provide another opportunity to contribute, but it was still vulnerable to capturing the views of the more engaged. It is not clear how generalisable the findings will be to other contexts.

These findings offer important lessons for large-scale improvement programmes. They show that the effort and time required to reach and implement an agreed approach to measurement for improvement, particularly when the measures are novel, should not be underestimated. Development of measurement systems requires cultural change and technical leadership. It is likely that at least 6 months is needed before an improvement programme starts to allow systems to be optimised. Even then, contestation about data definitions and symptoms about data collection burden may persist and should be anticipated. The collaborative model may have rich potential as a design and developmental phase in large-scale improvement programmes, but may not on its own produce change when external contexts are unfavourable.
